# Supplementary material for: Open diffusion magnetic resonance imaging and connectivity data for epilepsy and surgery: The IDEAS II release
Source: Epilepsia. 2026 Mar 7;67(6):2912–23. doi: 10.1002/epi.70186 (PMC13285235; doi:10.1002/epi.70186)
Supplement: Supplementary file 1 — Data S1. [file EPI-67-2912-s001.docx]

**Open diffusion MRI and connectivity data for epilepsy and surgery: The IDEAS II release**

# Supplementary

| **Table S1:** links to IDEAS II data* | |
| --- | --- |
| masks | <https://figshare.com/s/476b37fd883c14f50324> |
| raw_data | <https://figshare.com/s/4ec743d20cf1c41ed01d> |
| minimally_processed | <https://figshare.com/s/e8c80939dafc5eead4b1> |
| processed | <https://figshare.com/s/2dbae1fbfe72f7e66e1a> |
| tables | <https://figshare.com/s/802f11be0d93f0ad20ff> |

Raw data is also available at <https://openneuro.org/datasets/ds007401>

Further information is available at <https://www.cnnp-lab.com/ideas-data>


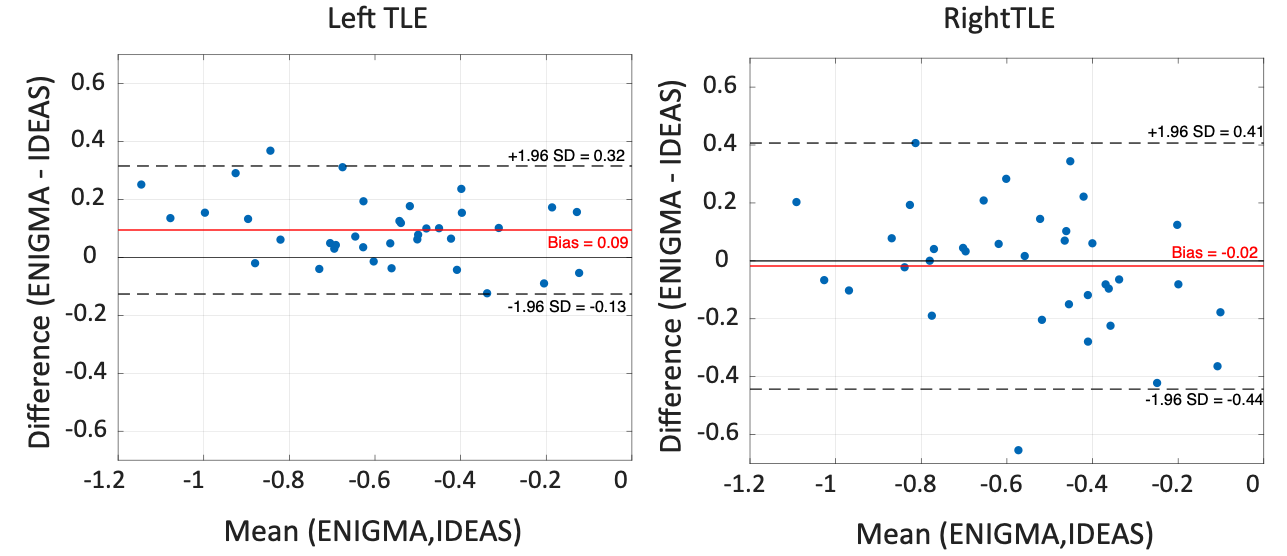


**Figure S1:** Bland-Altman plots for similarities between IDEAS II and ENIGMA findings.


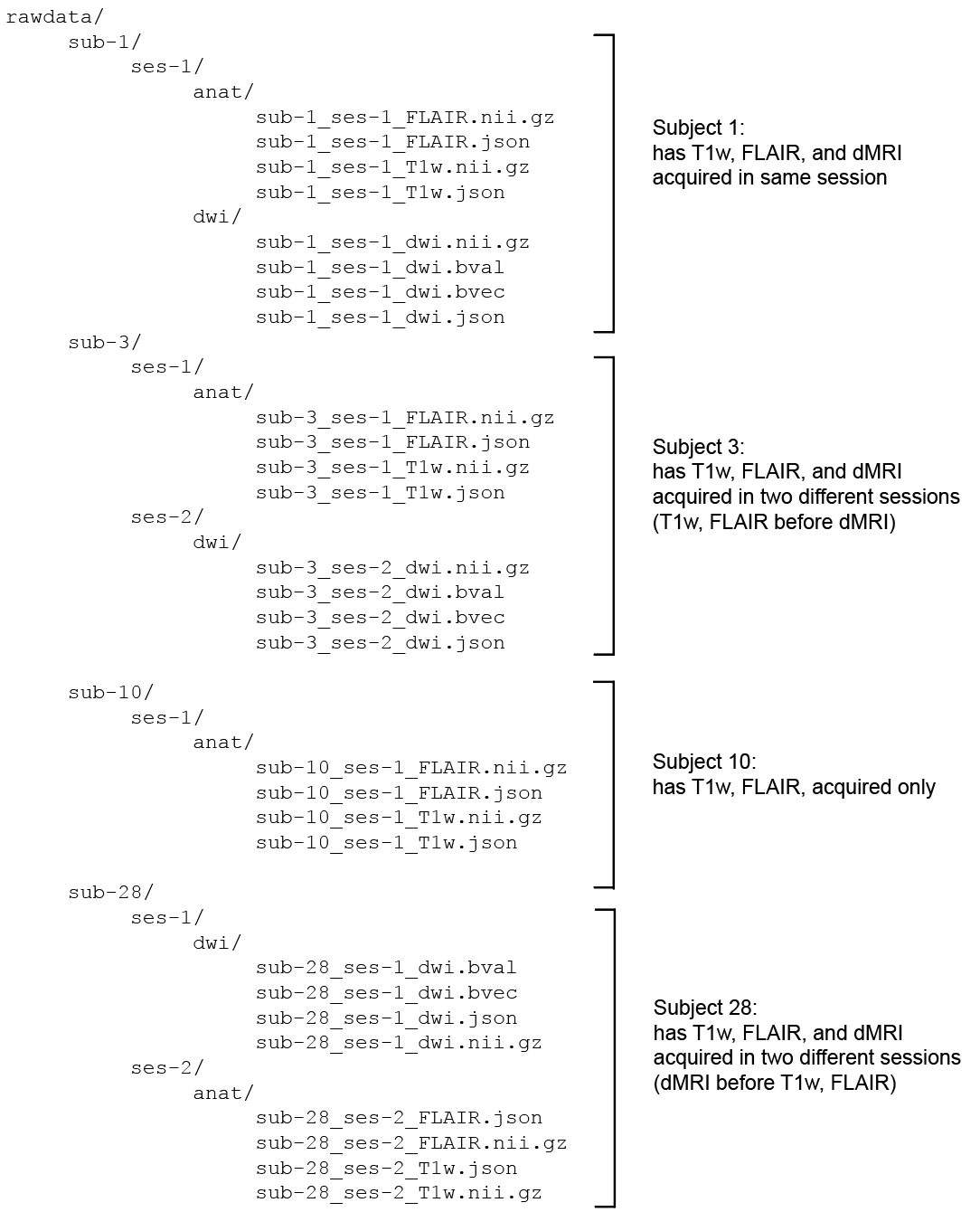


Figure S2: summary of BIDS data organisation of the four different scenarios. Note that all T1w and FLAIR scans are identical to those released already in IDEAS I.


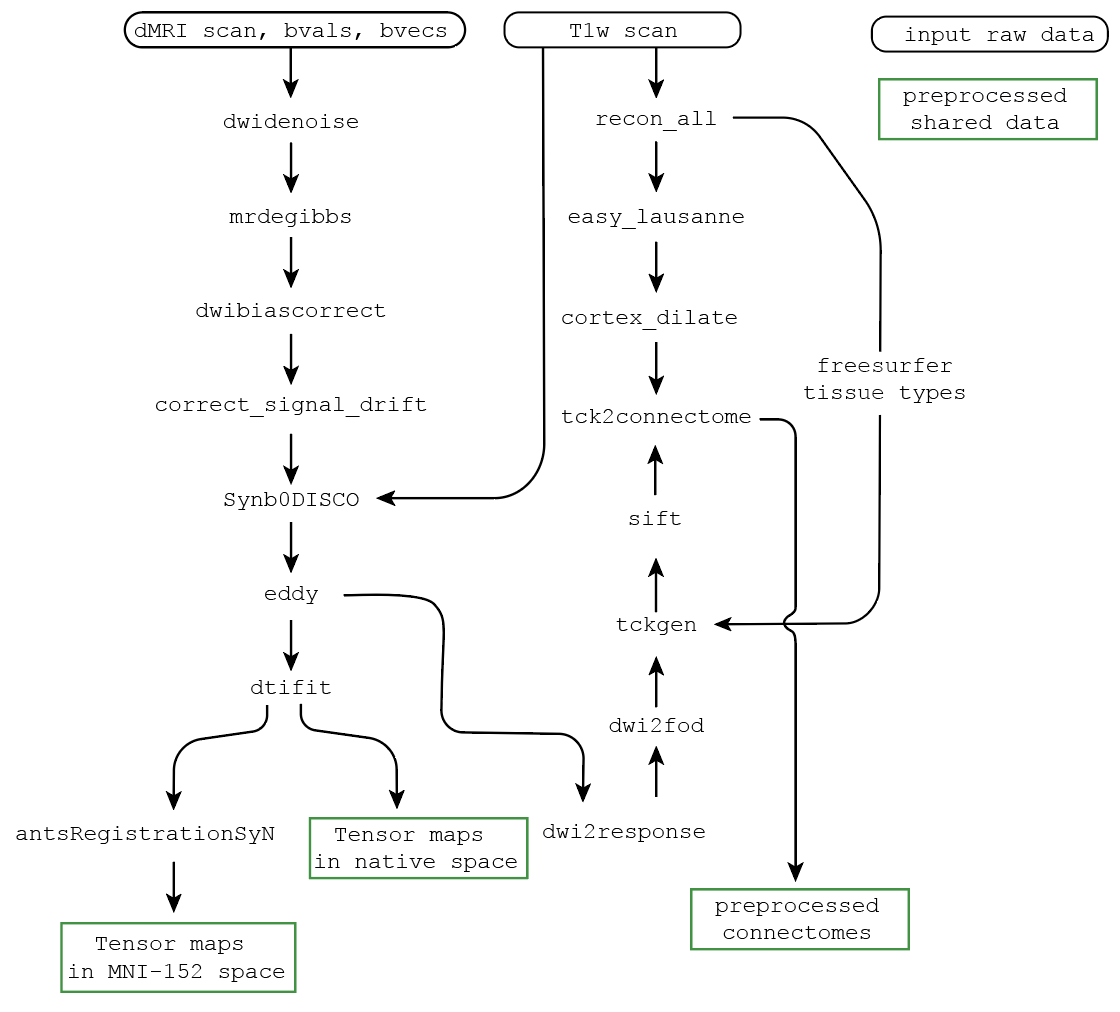


**Figure S3: Summary of processing steps.** All steps use publicly available pipelines. If the T1w and dMRI scan were acquired in different sessions, a modified version of this pipeline with linear registration first.

IDEAS II readme file

The release on OpenNeuro includes only raw T1w, FLAR and diffusion MRI scans. The key difference in the IDEAS II data, compared to IDEAS I is the addition of diffusion MRI. Fully processed data, including resection masks and other demographic information can be found at the following locations: <https://www.cnnp-lab.com/ideas-data> Below, is a unified list of all data available across the two IDEAS data releases.

- Raw T1w, FLAIR and diffusion MRI scans organised in BIDS format. Nifti and json descriptors included: <https://figshare.com/s/4ec743d20cf1c41ed01d>
- Resection masks in native space of the T1w scan. <https://figshare.com/s/476b37fd883c14f50324>
- Raw T1w and FLAIR data, additional to minimally processed diffusion MRI data. <https://figshare.com/s/e8c80939dafc5eead4b1>
- Raw T1w and FLAIR data, additional to fully processed diffusion MRI data. Fully processed data includes tensor maps in native space, MNI-152 space, and connectomes. <https://figshare.com/s/2dbae1fbfe72f7e66e1a>
- Freesurfer surface and volumetric reconstructions derived from the shared T1w scan <https://figshare.com/s/b13b8bb41390d3f7a088>
- Freesurfer thickness, volume, and surface areas for the Desikan-Kiliany parcellation <https://figshare.com/s/010142dd51e37ba4e4e2>
- Clinical and demographic metadata <https://figshare.com/s/bab70268afeb1071202b>
- Table indicating the percentage of each brain region in the Desikan-Kiliany atlas subsequently resected by surgery. <https://figshare.com/s/097ba0e254e36f0eee52>
- Freesurfer thickness, volume, and surface areas for the Desikan-Kiliany parcellation, z-scored against normative controls post-combat. <https://figshare.com/s/8c086fc295a75f85e628>

For updates please subscribe to the [mailing list](https://www.jiscmail.ac.uk/cgi-bin/wa-jisc.exe?SUBED1=IDEAS-DATA&A=1).

If you use T1w or FLAIR scans, please cite the following publication:

Taylor, Peter N., et al. "The imaging database for epilepsy and surgery (IDEAS)." *Epilepsia* 66.2 (2025): 471-481.

If you use the diffusion MRI scans, please cite the following publication:

Taylor, Peter N., et al. “Open diffusion MRI and connectivity data for epilepsy and surgery: The IDEAS II release.” Epilepsia [in press] (2026)

If you use the resection masks please cite the following publication:

Simpson, Callum., et al. “Automated generation of epilepsy surgery resection masks: The RAMPS pipeline” Imaging Neuroscience. (2025) 3 IMAG.a.147
